# A New Prognostic Sign in Jaundice

**Published:** 1855-09

**Authors:** Cathcart Lees

**Affiliations:** Physician to the Meath Hospital


A New Prognostic Sign in Jaundice. By Dr. Cathcart Lees, Physician to the Meath Hospital.—Many cases of jaundice have occurred, writes Dr. Lees, in which delirium, convulsions, and coma supervened, and proved rapidly fatal, although accurate examination failed to discover any mechanical obstacle to the passage of bile out of the system, the bile ducts being pervious and empty; so that this form of disease has been described as fatal jaundice from *suppressed secretion* of bile which means, that the jaundice in such cases depends on the retention in the blood of the elements of the bile, which in the healthy state is separated only, not formed at the liver, and which, when retained, acts on the nervous system nearly as a narcotic poison,—causing a condition of the system analogous to that occasioned by the suspension of the secretion of urine in cases of ischuria renalis, or in some cases of albuminuria. Dr. Alison explains this by supposing that the retention in the blood of matter destined to excretion is much more hurtful to the living body than the *reabsorption* into the blood of matters which have been excreted at their appropriate organs, but not discharged from the system in their natural way, owing to some mechanical obstruction in either the biliary or urinary passages. In reference to this class of cases, Dr. Budd, in his work on "Diseases of the Liver," after detailing some observations recorded by Alison, Bright, Graves, and others, thus writes: "It does not seem possible to deduce from the cases that have been related any sure means of distinguishing jaundice that results from suppressed secretion, from jaundice produced by temporary closure of the ducts, except in the particular cases where the jaundice immediately follows a powerful emotion, or occurs in the course of purulent phlebitis; or in consequence of known poisoning; or where, as in the instances related by Dr. Griffin and Dr. Hanlon, it occurs with peculiar characters in several members of a family, or in several persons living together, in succession. In all these instances, knowledge of the cause of the disease, or of some peculiar circumstances under which it may have arisen, gives significance to symptoms that would otherwise be vague and ambiguous. In other instances, where our judgment must be formed from the symptoms merely, the diagnosis is much more difficult." Now, it has struck me that the examination of the urine might be of some use in forming not only our diagnosis. but also our prognosis, in these cases of jaundice, particularly as in none of the cases recorded by the writers I have mentioned is there any analysis given of this secretion. I have, therefore, had the urine in some cases of jaundice carefully examined by competent persons lately, and shall proceed to detail one case, and give the results of two others nearly similar, in all of which an important principle of the bile was detected in the urine.

Dr. Lees then relates a case of extreme jaundice from retention of bile, the retention being due to obstruction of the ducts by cancerous growths; and he refers to two other cases of an analogous character, in which, notwithstanding the saturation of the system with bile, there was a complete absence of any symptoms referable to the nervous system. In these cases *cholic acid* was found in the urine; and it is to the excretion of this principle in this way that Dr. Lees is disposed to refer the absence of nervous symptoms. So far, however, this is a mere assumption, and no cases are given to show that, where the nervous symptoms are present, there has been an absence of cholic acid in the urine. Unfortunately, also, the means of verifying the presence of cholic acid are complicated, and we may yet have to wait some time for the required evidence.—[*Dublin Quar. Jour. of Med.*] *Half-Yearly Abstract of Medical Sciences.*

